# NL-EVDR: Netherlands—ExtraVascular Device Registry

**DOI:** 10.1007/s12471-023-01768-1

**Published:** 2023-03-02

**Authors:** Fleur V. Y. Tjong, Karel T. N. Breeman, Lucas V. A. Boersma, Sing-Chien Yap, Lieselot van Erven, Michelle D. van der Stoel, Vincent F. van Dijk, Alexander H. Maass, Reinoud E. Knops, NL-EVDR Working Group and NHR Device Registration Committee

**Affiliations:** 1 Department of Cardiology, Amsterdam University Medical Centres, location Academic Medical Centre; Amsterdam Cardiovascular Sciences, Heart Failure and Arrhythmias, Amsterdam, The Netherlands; 2 Department of Cardiology, St. Antonius Hospital, Nieuwegein, The Netherlands; 3 Department of Cardiology, Erasmus Medical Centre, Rotterdam, The Netherlands; 4 Department of Cardiology, Leiden University Medical Centre, Leiden, The Netherlands; 5 Netherlands Heart Registration, Utrecht, The Netherlands; 6 Department of Cardiology, University Medical Centre Groningen, Groningen, The Netherlands

**Keywords:** Extravascular, Leadless pacemaker, Subcutaneous implantable cardioverter-defibrillator, Registry

## Abstract

Cardiac implantable electronic device (CIED) therapy is an essential element in treating cardiac arrhythmias. Despite their benefits, conventional transvenous CIEDs are associated with a significant risk of mainly pocket- and lead-related complications. To overcome these complications, extravascular devices (EVDs), such as the subcutaneous implantable cardioverter-defibrillator and intracardiac leadless pacemaker, have been developed. In the near future, several other innovative EVDs will become available. However, it is difficult to evaluate EVDs in large studies because of high costs, lack of long-term follow-up, imprecise data or selected patient populations. To improve evaluation of these technologies, real-world, large-scale, long-term data are of utmost importance. A Dutch registry-based study seems to be a unique possibility for this goal due to early involvement of Dutch hospitals in novel CIEDs and an existing quality control infrastructure, the Netherlands Heart Registration (NHR). Therefore, we will soon start the Netherlands—ExtraVascular Device Registry (NL-EVDR), a Dutch nationwide registry with long-term follow-up of EVDs. The NL-EVDR will be incorporated in NHR’s device registry. Additional EVD-specific variables will be collected both retrospectively and prospectively. Hence, combining Dutch EVD data will provide highly relevant information on safety and efficacy. As a first step, a pilot project has started in selected centres in October 2022 to optimise data collection.

Cardiac implantable electronic device (CIED) therapy is an essential element of the contemporary treatment of cardiac arrhythmias [1, 2]. For many decades, all CIEDs consisted of a pulse generator placed in a subcutaneous/subpectoral pocket and a transvenous lead, which passed through the subclavian or cephalic vein to the endocardium. These so-called transvenous CIEDs are associated with substantial morbidity and mortality due to complications, such as pneumothorax and lead dysfunction, which are mainly attributable to the transvenous lead, and subsequent re-interventions [3, 4].

## The era of extravascular devices

To prevent lead-related complications, extravascular devices (EVD) have been developed over the past 15 years, such as the subcutaneous implantable cardioverter-defibrillator (S-ICD) and intracardiac leadless pacemaker (LP) [5–8]. To compare these new technologies with conventional transvenous CIEDs, a randomised controlled trial (RCT) is the preferred study design, but only few RCTs have been performed (PRAETORIAN, ATLAS [5, 9]). This type of study is often not feasible because of the large number of patients needed for sufficient power and the related costs and study duration. Observational (post-approval) registries can also provide valuable information but carry important limitations, such as low numbers of included patients, low precision of larger datasets with limited variables (e.g. insurance datasets) and the fact that they mostly contain acute data and lack long-term follow-up data. Additionally, industry-initiated studies often include a selected, healthier patient population than the everyday clinical practice population. To provide an enhanced evaluation of these technologies, real-world, large-scale, long-term data are of utmost importance.

Currently, we are still at an early stage of the EVD era. The most extensively studied EVDs are the S‑ICD and LP. The S‑ICD consists of a subcutaneous/intermuscular pulse generator and a subcutaneous lead with a shock coil parallel to left of the sternum. Safety and efficacy at up to 5 years follow-up have been demonstrated in large registries [10, 11]. In a previous RCT, the S‑ICD was proven non-inferior to the transvenous ICD with respect to all device-related complications over more than 4 years of follow-up, with fewer lead-related complications and systemic infections [5, 12]. The LP is a miniaturised pacemaker implanted via the femoral (or jugular) vein that is fully contained within in the heart. Currently, only a right ventricular LP is commercially available, which was proven safe and effective during up to 2 years of follow-up [13].

Other EVDs that have been developed and are currently being studied clinically are: extravascular ICD (EV-ICD), an ICD with a substernal epicardial lead; modular cardiac rhythm management (mCRM) system, consisting of an S‑ICD and LP that can wirelessly communicate to provide anti-tachycardia pacing; and dual-chamber LP, consisting of right atrial and right ventricular LPs that communicate wirelessly. Only preclinical or short-term data on these devices are available [14–16]. At this rate of development, many new EVDs will be available for clinical studies in the next decade. To be able to inform about long-term results and place these new EVDs in the current device armamentarium, a vendor-neutral, long-term registry of EVDs is required.

## Dutch national EVD registry

We hereby present the start of a nationwide registry with long-term follow-up: the Netherlands—ExtraVascular Device Registry (NL-EVDR). Being the Dutch national EVD registry, the NL-EVDR can meet all abovementioned requirements for a sustainable source of reliable information on the safety and efficacy of these new technologies. The NL-EVDR will be incorporated in the Netherlands Heart Registration (NHR), a quality and registration organisation that collects information about all cardiac procedures and short-term follow-up (up to 90 days) in the Netherlands as a means of quality control. Physicians who are mandated by their hospital to instruct the NHR to process their data are united in registration committees. The NHR Device Registration Committee has determined a standard set of variables and manages these data about CIEDs. The Netherlands Society of Cardiology (*NVVC*) has defined which elements are mandatory as part of their quality policy.

The NL-EVDR will be organised as an add-on to the device registry, with additional EVD-specific information and long-term follow-up. As there already is an infrastructure for data collection with a high degree of completeness of the data, the registry will adequately represent a real-world cohort. Furthermore, information about 3100 EVDs is currently available. This emphasises the popularity of EVDs in the Netherlands, and possibly, additional information about these devices can be collected retrospectively. Dutch hospitals have been involved in many of the early EVD studies, and presently, clinical studies with the mCRM system and dual-chamber LP are led by a Dutch hospital [14, 17–20]. Hence, combining Dutch data on EVDs will provide a unique opportunity to describe long-term safety and efficacy.

**Fig. 1:**
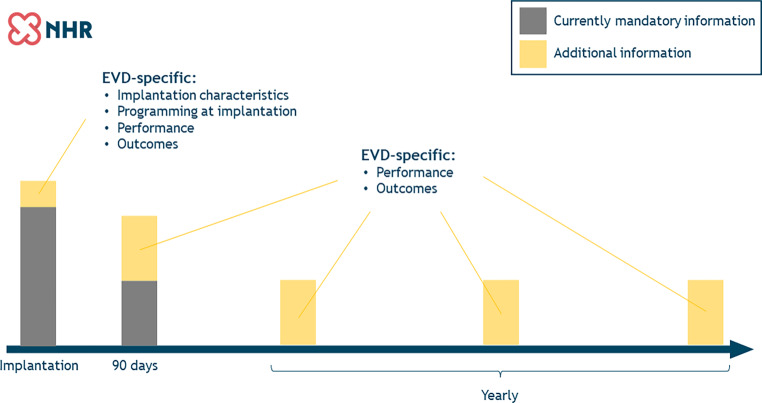
Overview of data collection in the Netherlands—ExtraVascular Device Registry. Collected data consist of currently mandatory information (*grey*) and additional, extravascular device (*EVD*)-specific information (*yellow*)

The collected variables in the NL-EVDR will encompass the already existing variable set, which has been defined as mandatory by the NVVC, as well as additional information (Fig. 1). The mandatory variables currently collected include patient and intervention characteristics and outcomes up to 90 days after implantation. When a patient receives a second device, the indication for this device is also noted. The additional EVD variables will be extra EVD-specific variables at implantation and information regarding complications and device performance at yearly follow-up visits. Moreover, EVD-specific complications will be added. In case of mortality, it will be noted whether the death was device-related.

The aim is to collect data both retrospectively and prospectively. Retrospective data will be obtained using a data collection sheet in collaboration with hospital data managers, while prospective data will be collected simultaneously with the already existing variable set. Eventually, this could be embedded in the NHR online environment (‘MijnNHR’). However, first, a pilot project will be conducted to evaluate the quality of the retrospective data collected in selected centres, ameliorate the prospective data collection process and evaluate the feasibility of additional data collection. If deemed feasible, data collection will be expanded to all Dutch PM/ICD centres. The pilot project has started in October 2022.
